# Computational Psychometrics for the Measurement of Collaborative Problem Solving Skills

**DOI:** 10.3389/fpsyg.2017.02029

**Published:** 2017-11-29

**Authors:** Stephen T. Polyak, Alina A. von Davier, Kurt Peterschmidt

**Affiliations:** ACTNext, ACT, Inc., Iowa City, IA, United States

**Keywords:** psychometrics, problem-solving, collaboration, clustering, simulation, game, evidence-centered

## Abstract

This paper describes a psychometrically-based approach to the measurement of collaborative problem solving skills, by mining and classifying behavioral data both in real-time and in post-game analyses. The data were collected from a sample of middle school children who interacted with a game-like, online simulation of collaborative problem solving tasks. In this simulation, a user is required to collaborate with a virtual agent to solve a series of tasks within a first-person maze environment. The tasks were developed following the psychometric principles of Evidence Centered Design (ECD) and are aligned with the Holistic Framework developed by ACT. The analyses presented in this paper are an application of an emerging discipline called computational psychometrics which is growing out of traditional psychometrics and incorporates techniques from educational data mining, machine learning and other computer/cognitive science fields. In the real-time analysis, our aim was to start with limited knowledge of skill mastery, and then demonstrate a form of continuous Bayesian evidence tracing that updates sub-skill level probabilities as new conversation flow event evidence is presented. This is performed using Bayes' rule and conversation item conditional probability tables. The items are polytomous and each response option has been tagged with a skill at a performance level. In our post-game analysis, our goal was to discover unique gameplay profiles by performing a cluster analysis of user's sub-skill performance scores based on their patterns of selected dialog responses.

## 1. Introduction

Collaborative problem solving (CPS) is considered as one of the critical skills for academic and career success in the twenty-first century (Griffin et al., 2012). The literature on this topic highlights changing trends that are leading to more employment opportunities that demand collaboration and interaction between people in problem-solving contexts (He et al., 2017; Oliveri et al., 2017). This trend has increased the need in the education industry to address ways to teach and assess these skills (von Davier et al., 2017). In this paper we consider the cognitive and social perspectives of the collaborative problem solving process and examine the circumstances under which collaborative problem solving might best take place to evaluate a participant's level of competency. We outline a structure through which the contributing processes can be monitored and assessed in an electronic environment. In doing so, we reference an emerging discipline called computational psychometrics that is growing out of traditional psychometrics and incorporates techniques from educational data mining, machine learning and other computer/cognitive science fields. We also introduce our initial work on a collaborative problem solving simulation in which a user is required to collaborate with a virtual agent in order to solve a series of tasks/problems within a first-person maze environment. We demonstrate two techniques based on our knowledge of computational psychometrics:

- Real-time Bayesian evidence tracing that updates sub-skill level probabilities as new evidence is presented.
- A post-game clustering analysis of a user's sub-skill performance scores aimed at defining different profiles of simulation results.

## 2. Materials and methods

In this section we share our study approach, starting with the identification and selection of the specific CPS sub-skills we monitored. We then describe our simulation/game design, task development and the construction of the conversation tree for the computer agent. Given these constructs, we detail our methods for computational psychometric evidence tagging and continuous evidence tracing. We overview the steps in study execution and data collection. Finally, we define our postgame analysis process that utilizes a set of machine-learning based clustering techniques.

### 2.1. CPS sub-skills

For this study, our methodology was to first select a set of collaborative problem solving sub-skills that have been researched and published as part of ACT's investigations into helping people achieve education and workplace success. In “Beyond Academics: A Holistic Framework for Enhancing Education and Workplace Success,” (Camara et al., 2015) identified facets beyond the well known core academic skills which include the domain-specific knowledge and skills necessary to perform essential tasks in the core content areas of English language arts, mathematics, and science. These additional areas include:

- Cross-cutting capabilities: General knowledge and skills necessary to perform essential tasks across academic content areas. This includes technology and information literacy, **collaborative problem solving**, thinking and metacognition, and studying and learning.
- Behavioral skills: The interpersonal, self-regulatory, and task-related behaviors important for adaptation to, and successful performance in, education and workplace settings.
- Education and career navigation skills: The personal characteristics, processes, and knowledge that influence individuals as they navigate their educational and career paths (e.g., make informed, personally relevant decisions; develop actionable, achievable plans).

As seen above, the cross-cutting capabilities section of the Holistic Framework includes collaborative problem solving as part of a broad, four category enumeration:

1. Technology and Information Literacy
2. **Collaborative Problem Solving**
3. Thinking and Metacognition
4. Studying and Learning

Within the framework, CPS skills are further decomposed into various sub-skills and sub-skill areas. For example, sub-skill areas within CPS include:

- Behavior
- Collaborative Communication
- Problem Analysis
- Solution Planning
- Extended Collaboration (Teamwork)

For this study, we selected 5 sub-skills to gather and analyze for CPS evidence:

- Feature Identification (FI): Identifies the key features of the problem space
- Maintaining a Shared Understanding (MU): Identifying and reconciling gaps in understanding
- Engagement/Interaction (EN): Engagement in the group process and the degree to which that engagement is self-initiated
- Strategy (S): Evidence of establishing a plan of action or policy designed to achieve a major or overall aim
- Evaluate (EV): Recognizing own strengths and weaknesses in relation to others.

#### 2.1.1. Sub-skill expected use

Obtaining an assessment at the sub-skill level provides granular evidence to fill in a portion of the Holistic Framework representation of the participant. This diagnostic information can be used to direct toward targeted resources or other remediation steps. It can also be used to provide a representative view of a participant's ability.

#### 2.1.2. CPS assessments

Society needs assessments that reflect the way people actually teach, learn and work. There are several examples of initiatives and assessments which pioneered a large-scale approach toward measuring CPS skills. These include:

- The Programme for International Student Assessment (PISA) 2015 administered a test of collaborative skills (PISA, OECD, 2013).
- The National Center for Educational Statistics (NCES) commissioned a white paper on the considerations for introduction of CPS in the National Assessment of Educational Progress (NAEP) (NCES, 2017).
- An edited interdisciplinary volume on innovative assessments of collaboration was just published with Springer Verlag (von Davier et al., 2017).
- A special issue of the Journal of Educational Measurement highlighting recent advances in measurement and assessment of cognitive and non-cognitive skills for both individuals and teams, and innoative ways of studying collaboration in education (von Davier, 2017).
- The Smarter Balance Consortium developed an assessment system where performance tasks, including collaborative tasks, are being considered for administration to students as a preparatory experience and are then followed with an individual assessment (Davey et al., 2015).

CPS skills are important for education and career success, but they are difficult to measure. Because CPS is largely enacted as an interactive set of tasks with partners, we need a means to provide a multi-agent setting in which the subjects under assessment can express their abilities. This means providing the opportunity to display the skills in a CPS task for discussion, negotiation, decision making, etc. with another participant, be they a human or simulated agent. In either case, all of these interactive data are referred to as “process data” that offer insight into the interactional dynamics of team members; they are relevant for defining collaborative tasks and for evaluating the results of the collaboration. In the past, these data were not available to scientists at scale. With advances in technology, these complex data can be captured in computerized log files and hence, may allow for meaningful inferences.

The process data from CPS tasks consist of time-stamped sequences of events. From a statistical perspective, these data are time series logs describing the actions and interactions of the users. See Hao et al. (2016) for a discussion of the CPS data. In addition to the process data, if the collaboration is set up in a cognitive (say, math) task, it will also result in outcome data. These types of data are more similar to the outcome data from the traditional tests and indicate if a particular question was answered correctly, or whether the problem was solved (and to what degree it was solved).

Attempting to measure collaboration using a game or other virtual environment is not novel. Neither are the ideas of stealth assessment (Shute et al., 2008b) or evidence centered assessment design (Mislevy et al., 2003; Shute et al., 2008b). However, it is still common to see measurement of collaboration provided by *post hoc* survey data collection (Sánchez and Olivares, 2011; Sung and Hwang, 2013). Measuring through in game data collection techniques holds value, in that more real-time determinations can be made and some of the disadvantages of self-reports (Paulhus et al., 2007) can be avoided, such as self-presentation (Robins and John, 1997).

### 2.2. Simulation/game design

In order to collect data and test hypotheses for this study, ACT developed a CPS game called “Circuit Runner”^1^ which allows subjects to play online, in a web browser, with the mission to solve a series of challenges in order to “win” the game. The player needs to collaborate with an automated, virtual agent that has information required to complete the challenges.

**Figure 1:**
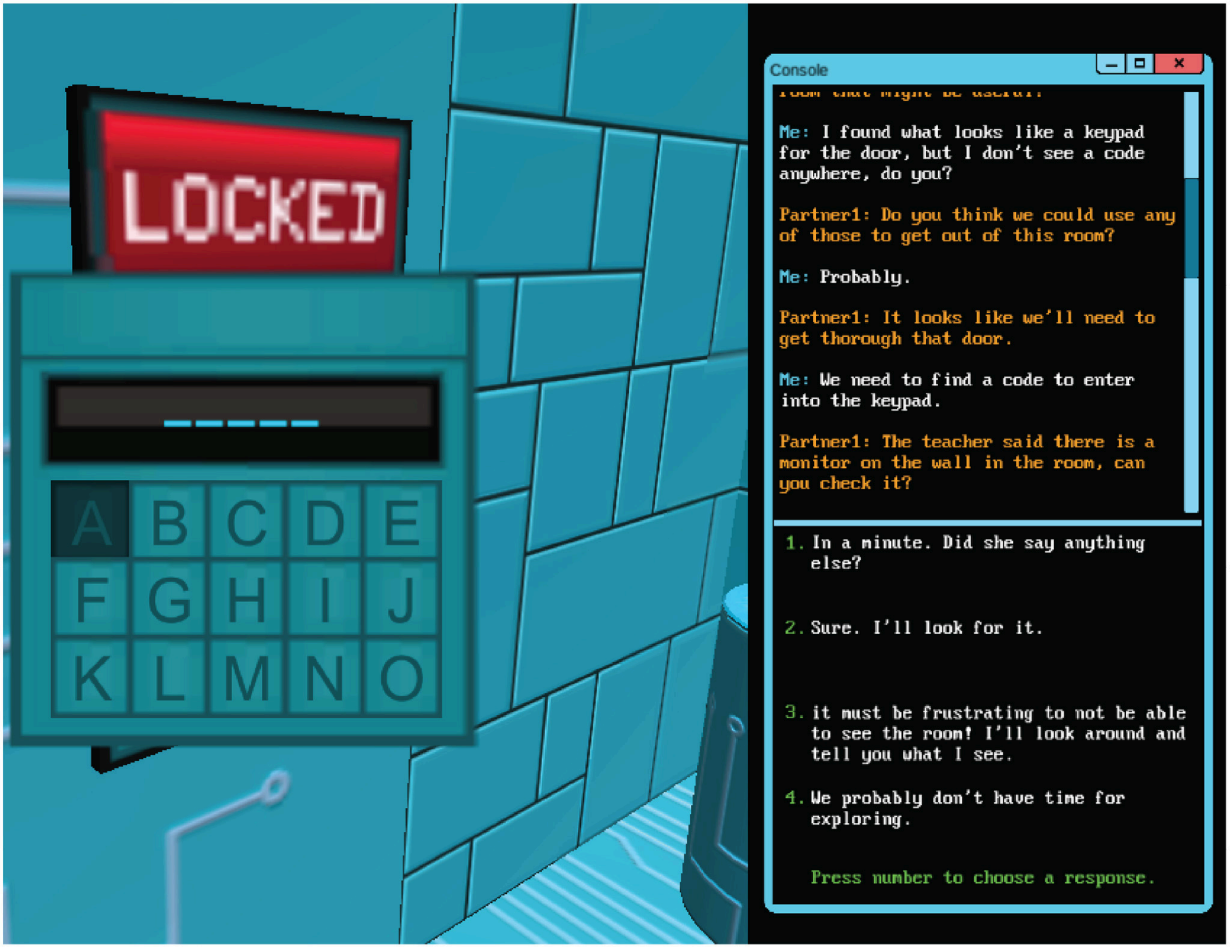
Circuit runner: a CPS dialog panel game screenshot.

In total there are five distinct challenges that range from an agent/player feature discussion around a coded, door-lock panel to a more sophisticated challenge that involves collaborative discovery of a sequence of power transfer steps in order to succeed. The player navigates from challenge to challenge via a 3-D maze in a first person perspective and is also given continuous access to the agent via a dialog panel which can present prompts and dialog responses from various dialog/conversation trees the player may select. A view of the conversation panel within the game is provided in Figure 1. All of the dialog response selections made by the player are recorded in a game “conversation flow” log data file. We can think of the presentation of conversation prompts via the agent as analogous to the presentation of item prompts in a more conventional assessment. The selection of conversation choices by the participant result in item responses captured during the game. Additional telemetry data is gathered including clicks, keystrokes, distance travelled, challenge duration, and dialog selection timing.

### 2.3. Design limitations

The canned responses of the dialog tree are a limitation of the current game design. For a more authenticate, natural flow a future design would allow for free text entry or potentially spoken dialog (using speech-to-text to obtain a machine-readable form of that input). Natural language processing (NLP) could then be used to help categorize and ultimately score a given response.

Interaction only occurring between one participant and a virtual agent is another limitation. Allowing multi-human to agent collaboration would add realistic variability and additionally provide another vector of evidence to observe demonstrated CPS skills via human to human interaction.

The game as well as the Holistic Framework's CPS section can be viewed as ultimately stemming from Steiner's proposal regarding group productivity (Steiner, 1972) and a future version that does allow for multi-human interaction would further envelope his proposed performance of a group on a task depended on three factors: the resources available to the group, the requirements of a given task, and the processes by which the group uses to solve a given task. Additionaly, the construction of the CPS section of the Holistic Framework looks to Camara et al. (2015) “operationalize the broader construct of collaboration and group work in order to identify specific cognitive skills and strategies that can improve performance.” (p. 23)

### 2.4. Computational psychometrics

Given these constructs for assessing CPS skills, we consider our methodological basis applying computational psychometrics (von Davier, 2015; von Davier et al., 2017). Computational psychometrics (CP) is defined as a blend of data-driven computer science methods (machine learning and data mining, in particular), stochastic theory, and theory-driven psychometrics in order to measure latent abilities in real-time.

This mixture of disciplines can also be formalized as iterative and adaptive hierarchical algorithms embedded in a theoretical psychometric framework. A similar hierarchical approach to multimodal data was discussed in Khan et al. (2013) and Khan (2015). In a computational psychometrics framework, the test development process and data analysis are rooted in test theory and start with the application of the principle of Evidence Centered Design (ECD) (Mislevy et al., 2006); then, the test is administered as a pilot and the (multimodal) fine grain data are collected along with the data from test items (e.g., multiple choice items). This approach is sometimes called a top-down approach because it relies on the expert-based theories. The next step involves a bottom-up approach, in which the data are analyzed by data mining and machine learning algorithms. If new relevant patterns are discovered in the data, these may be incorporated in the revised psychometric models. Next, the psychometric models are revised and the process is repeated with a second round of data collection. One may also apply stochastic processes to the process data. Once the psychometric model is defined and the estimation of the model parameters is stable, the assessment is administered to the population of interest. On the operational data, only supervised machine learning algorithms and already defined and validated psychometric models are further used in order to achieve a stable measurement and classification rules.

This framework involves designing the system (learning and/or assessment) based on theory, identifying constructs associated with the competency of interest, and finding evidence for these constructs from the process data, including video or audio data (Bazaldua et al., 2015). The need for an expansion of the psychometrics framework to include data-driven methods occurred due to the characteristics of the data (dependencies, fine grain size, and sheer volume).

The types of psychometrics models associated with complex data with dependencies have primarily been Bayesian Belief Networks (BBN) (Levy, 2014; Mislevy et al., 2014). BBNs model the probability that a student has mastered a specific knowledge component, conditional on the sequence of responses given to previous elements of a task and eventually to other tasks, whether they are associated with that knowledge component or not (as long as they are part of the network and share at least an indirect connection. BBNs have been applied in games to represent student knowledge and thereby guide the activities of the tutoring system (Corbett and Anderson, 1994; Shute et al., 2008a; VanLehn, 2008; Desmarais and Baker, 2012). BBNs seem attractive for measuring CPS skills, but they have not been adapted to represent the knowledge of multiple individuals simultaneously.

There are stochastic models (point processes, for example) that can be used to model the temporal dynamics of the CPS tasks (von Davier and Halpin, 2013), or hidden Markov models (Soller and Stevens, 2007); there are also models based on the cognitive or social theories such as Agent-based modeling (Bergner et al., 2015) and Markov Decision Process, which is a cognitive model with parameters that describe the goals or beliefs of the agents and which defines behavior as an optimization of expected rewards based on current beliefs about the world (LaMar, 2014). With the aid of data mining techniques we may reduce the dimensionality of the dataset by extracting interpretable patterns which allow research questions to be addressed that would otherwise not be feasible (Romero et al., 2009). This process may help in the scoring process, by assigning different scores to different clusters. Recent papers illustrate the identification of new evidence to revise the psychometric models (Kerr and Chung, 2012; Kerr, 2015; Zhang et al., 2015).

For the past decade, machine learning algorithms have been used in education to automatically grade written essays; in order to automatically grade and interpret the speech and chat in collaborative interactions we are using similar algorithms; similarly, we can use machine learning for the automatic detection of emotions or affective states during collaboration (Khan, 2015; von Davier et al., 2016).

Bringing the rigor and advanced analysis techniques represented by CP to assess skills that are considered “soft” or otherwise lacking a traditional structure (ie. an acedemic assessment like mathematics), evolve the field. Creating a process to provide for repeatability as well as reducing bias from *ad hoc* methodologies are both desired outcomes of using CP.

In specific practical applications of CP, this hierarchical inference data model may be implemented in simplified or less explicit forms.

#### 2.4.1. Skill evidence tagging

**Figure 2:**
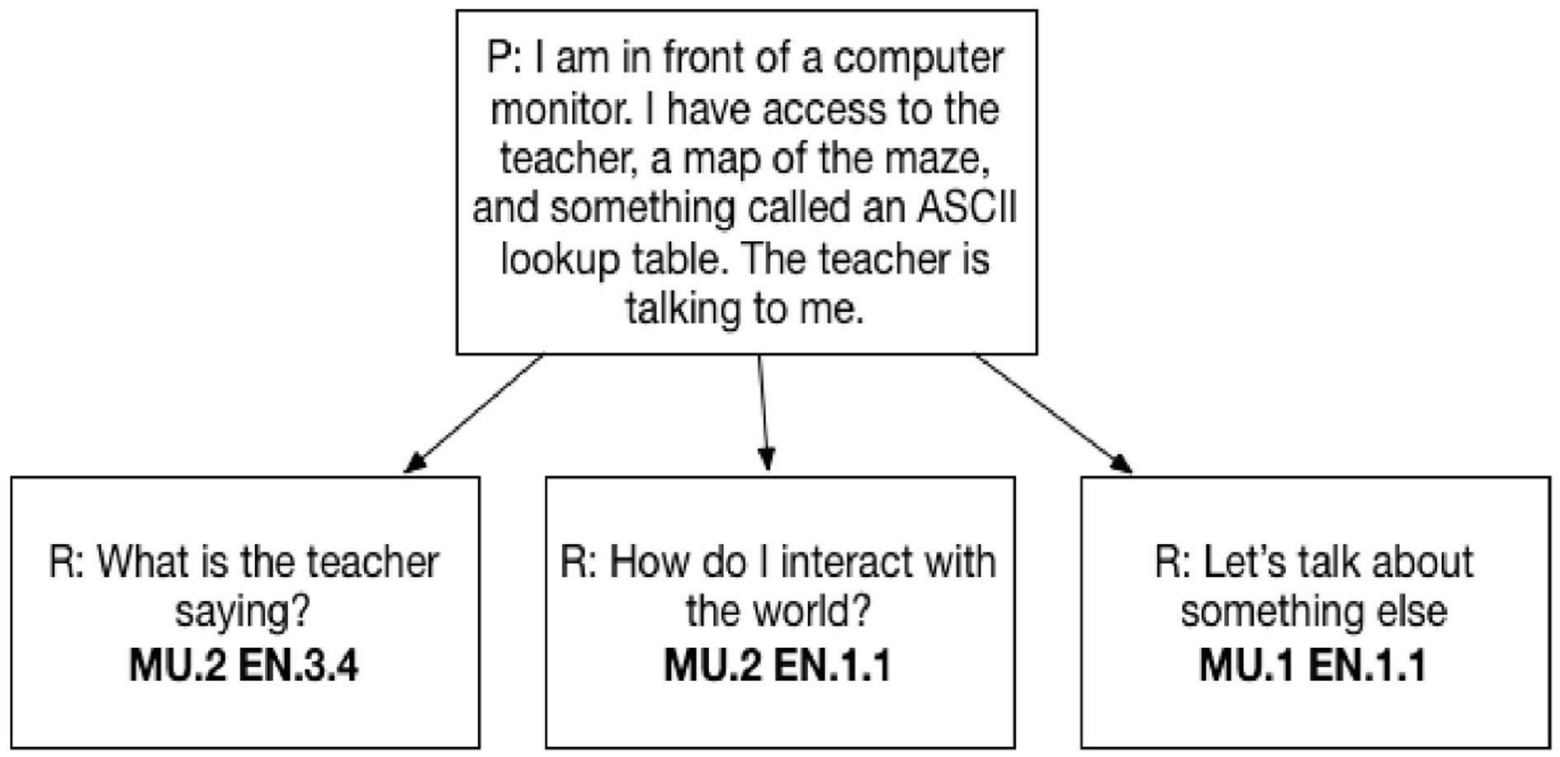
CPS response coding.

For the “Circuit Runner” game, ACT holistic framework researchers designed the tasks and the potential conversation flows, so that they would require participants to collaborate with the virtual agent in a way that would provide evidence of their latent skill ability associated with our selected CPS sub-skills. The dialog tree responses were tagged with one or more sub-skills that were expert judged to provide skill evidence. Furthermore, this evidence was also refined into a level tag using a 3 level enumeration of High, Med, and Low. This was completed by the work of subject matter experts and aligned with the standards set forth in the Holistic Framework (Camara et al., 2015). The experts then verified and approved this tagging and leveling based on committee agreement. The entirety of the tree and the tagging will not be completely disclosed for full reproducibility due to the intellectual property of the dialog tree as well as the proprietary means by which a content expert tagged and developed them. In Figure 2 we illustrate this tagging for one item/dialog tree prompt:

“I am in front of a computer monitor. I have access to the teacher, a map of the maze, and something called an ASCII lookup table. The teacher is talking to me.”

and a selected dialog response of:

“What is the teacher saying?”

This participant event/action presents evidence of CPS skills:

- Monitoring Understanding (MU) at the Med (.2) level (MU.2)
- Engagement (EN) at a High (.3.x) level (EN.3.4)

These items are polytomous and can effectively be scored for a participant based on their sub-skill association and level identification.

##### 2.4.1.1. Bayesian evidence tracing

We can see that conversation flow between the participant and agent provides us with a continuous stream of evidence of a participant's CPS sub-skill, our research question was:

“Given the real-time, sequential evidence presented via the data of dialog response selections in this game, can we intelligently predict the performance level at each sub-skill?”

The methodology we chose to follow to answer this question used a Bayesian approach related to those typically found in intelligent tutoring systems, such as Bayesian Knowledge Tracing (BKT) (Corbett and Anderson, 1994). The steps to demonstrate this were as follows:

- Extract raw conversation flow game log from a set of played games
- Transform the conversation flow into a flattened file that combines prompt and response and filter out any potential test data
- Generate a 1-Hot encoding of evidence (discussed below)
- Compute Bayesian predictions for all five sub-skills, across each performance level
- Plot the evidence tracing for insight/analysis.

##### 2.4.1.2. Extract

The log data file extracted from the game is outlined in Table 1. Each user can have 1 or more sessions and each session can have 1 or more games. In practice though we are typically only interested in 1 game for a single user. As we can see, the log collects the presentation of a dialog tree prompt to the user in a game as row type “P.” The prompt presented is recorded in the column “prompt_id.” Row type “R” records the response selected by the user in the game for the prompt row immediately preceding it in the log. This raw game log file contained the game session log for several game instances.

**Table 1:** Log file format.

**Session_id**	**User_id**	**Game_id**	**Time**	**Type**	**Prompt_id**	**Response**	**…**
19	11	1	2015-09-28T15:29:39.302222	P	0.1		
19	11	1	2015-09-28T15:29:49.627254	R		2	
19	11	1	2015-09-28T15:29:49.627254	P	0.3		
19	11	1	2015-09-28T15:29:50.906382	R		2	
…							

##### 2.4.1.3. Transform

Our next step was to flatten this representation so that the prompt and the response rows were combined into a single record. Additionally, we also filtered out data rows that were known to be developer gameplay “user_ids” so that we were only looking at data from actual subject participants. There were also prompt rows followed by some in game action. Instead of a response to that prompt, the user had done something that subsequently caused another prompt to appear. Since there was no response to that initial prompt, it, along with the following action, were also filtered out. Ultimately, *N* = 159 unique games for this analysis.

##### 2.4.1.4. 1-Hot

Taking the flattened prompt/response data, we encoded each game as a single row in a 159x286 matrix. The number of rows is the N count and the number of columns are the three identifiers (session, user, game) plus the 283 potential, selectable dialog responses (*D* = 283). We encoded a “1” if the user selected the identified response at any time during the game. It should be noted that several of the dialog sub-trees can allow a user to loop back through the tree within a single game. If the user selected a particular response more than once in a game we still recorded the selection with a single “1.” Otherwise, if the user never selected a particular response during the game the encoding for that column is “0.”

##### 2.4.1.5. Compute

Before we introduce our computation of probabilities for the performance levels of a game's CPS sub-skills, let's first review Bayes' theorem and how its application will allow us to trace the evidence over time.

*2.4.1.5.1. Bayes theorem*. One way to think of Bayes' theorem (Bayes and Price, 1763) is that it gives us a way to update the probability of a hypothesis, H, in light of some body of evidence, E. This way of thinking about Bayes' theorem is called the diachronic interpretation. More precisely, the probability of the hypotheses changes over time as we see new evidence. Rewriting Bayes' theorem with H and E yields

(1) $$p(H|E)=\frac{p(E|H)p(H)}{p(E)}$$

In this interpretation, each term has a name:

- *p*(*H*) is the probability of the hypothesis before we see the evidence, called the prior probability, or just “prior.”
- *p*(*H*|*E*) is what we want to compute, the probability of the hypothesis after we see the evidence, called the “posterior.”
- *p*(*E*|*H*) is the probability of the evidence under the hypothesis, called the likelihood.
- *p*(*E*) is the probability of the evidence under any hypothesis, called the normalizing constant.

**Figure 3:**
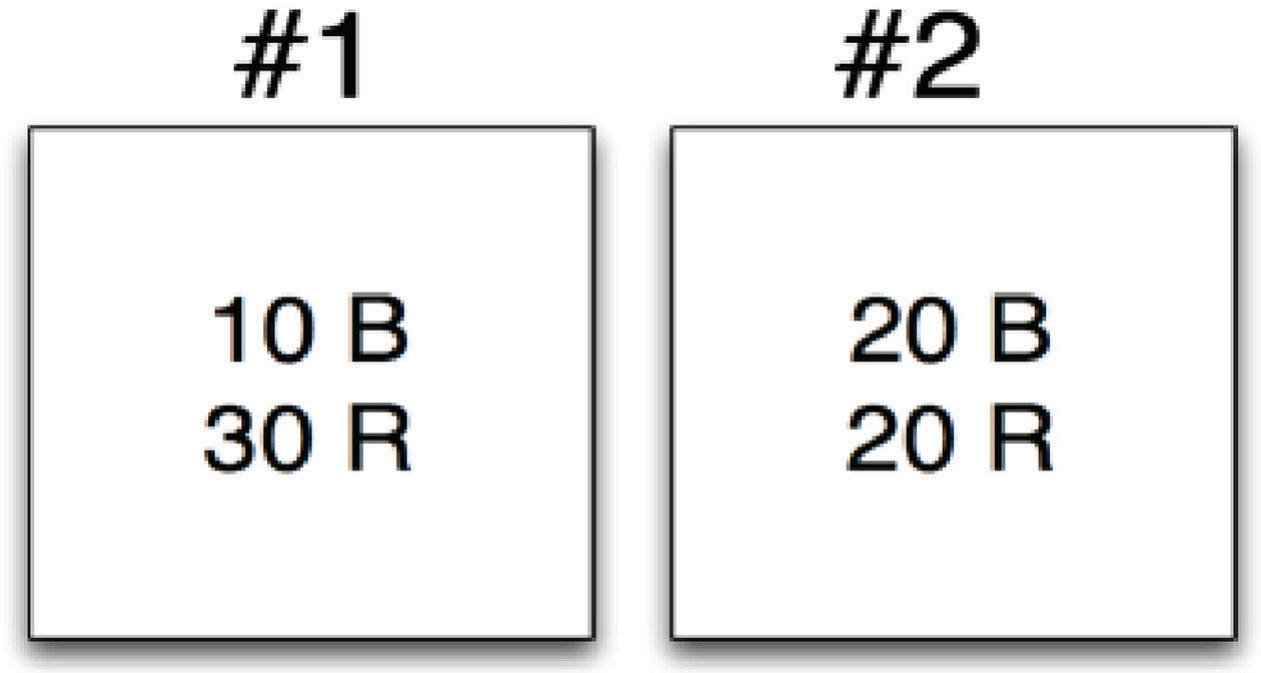
Bayesian selection example.

As an example, let's consider an application of Bayes' Theorem to a simple selection task using two bins to select from. On the performance of this task, we will consider the evidence (E) from a selection event and attempt to compute the probability of two competing hypotheses (*H*_1_) and (*H*_2_). Hypothesis 1 will consider that the selection event happened using bin 1 and hypothesis 2 will consider that the event used bin 2. In Figure 3 we depict the two bins, bin #1 and bin #2. Bin #1 contains 10 blue widgets (B) and 30 red widgets (R). Bin #2 contains 20 blue widgets (B) and 20 red widgets (R). Let's say that a selection event occurs and the evidence is that of a red widget (R). We will now apply the Bayes' theorem to consider the probability associated with each hypothesis:

1. *H*_1_: The red widget came from bin #1
2. *H*_2_: The red widget came from bin #2

The prior for both *p*(*H*_1_) and *p*(*H*_2_) are the same, $\frac{1}{2}$, because we are assuming that red and blue widgets appear equally in each bin. The likelihoods are different though, as we can see based on the composition of the bins. Specifically, we have

(2) $$p(E|H_{1})=\frac{3}{4}$$

(3) $$p(E|H_{2})=\frac{1}{2}$$

Putting this all together we can compute the posterior for both hypotheses as:

(4) $$p(H_{1}|E)=\frac{\frac{1}{2}*\frac{3}{4}}{\left(\frac{1}{2}*\frac{3}{4})+(\frac{1}{2}*\frac{1}{2}\right)}=0.6$$

(5) $$p(H_{2}|E)=\frac{\frac{1}{2}*\frac{1}{2}}{\left(\frac{1}{2}*\frac{3}{4})+(\frac{1}{2}*\frac{1}{2}\right)}=0.4$$

We can then state that given the evidence of a red widget we believe there is a 60% chance this was associated with bin #1 and a 40% chance this was associated with bin #2.

##### 2.4.1.6. Response to skill

Given this computation, we can apply it to the evidence and hypotheses we have for the CPS game. In our selection example, the evidence was straight-forward: was the widget blue or red? In the CPS game we need a lookup table for our response to determine which CPS sub-skill and at which performance level the response selection evidence is associated with. The first column of the lookup table combines a prompt identifier and the response, i.e., “0.1–1” (the following row then containing “0.1–2” for the second response of this prompt). The second column contains the noting of skills and levels such as “EN.3.4:FI.2.2:MU.2,” that has been tagged by an ACT content expert as providing evidence of:

- Engagement (EN) at a high level (3) (and specifically explanation #4 in that high level)
- Finding Information (FI) at a med level (2) (and specifically explanation #2 in that med level)
- Monitoring Understanding (MU) at a med level (2)

As Mislevy et al. (2014) describe in their application of ECD to interpreting game log data, we can refer to these sub-skills as latent variables, student model variables (SMVs) or competencies/proficiencies and will denote them using θ, “[the authors] posit that students' performances, characterized by features *x*_*j*_, arise from some underlying dimensions of knowledge, skill, familiarity, preferences, strategy availabilities, or whatever way we want to characterize them for the purposes at hand. These are called latent variables in the psychometric literature, and student model variables (SMVs), or sometimes competencies or proficiencies, in ECD terminology. We will denote them by θ”

**Figure 4:**
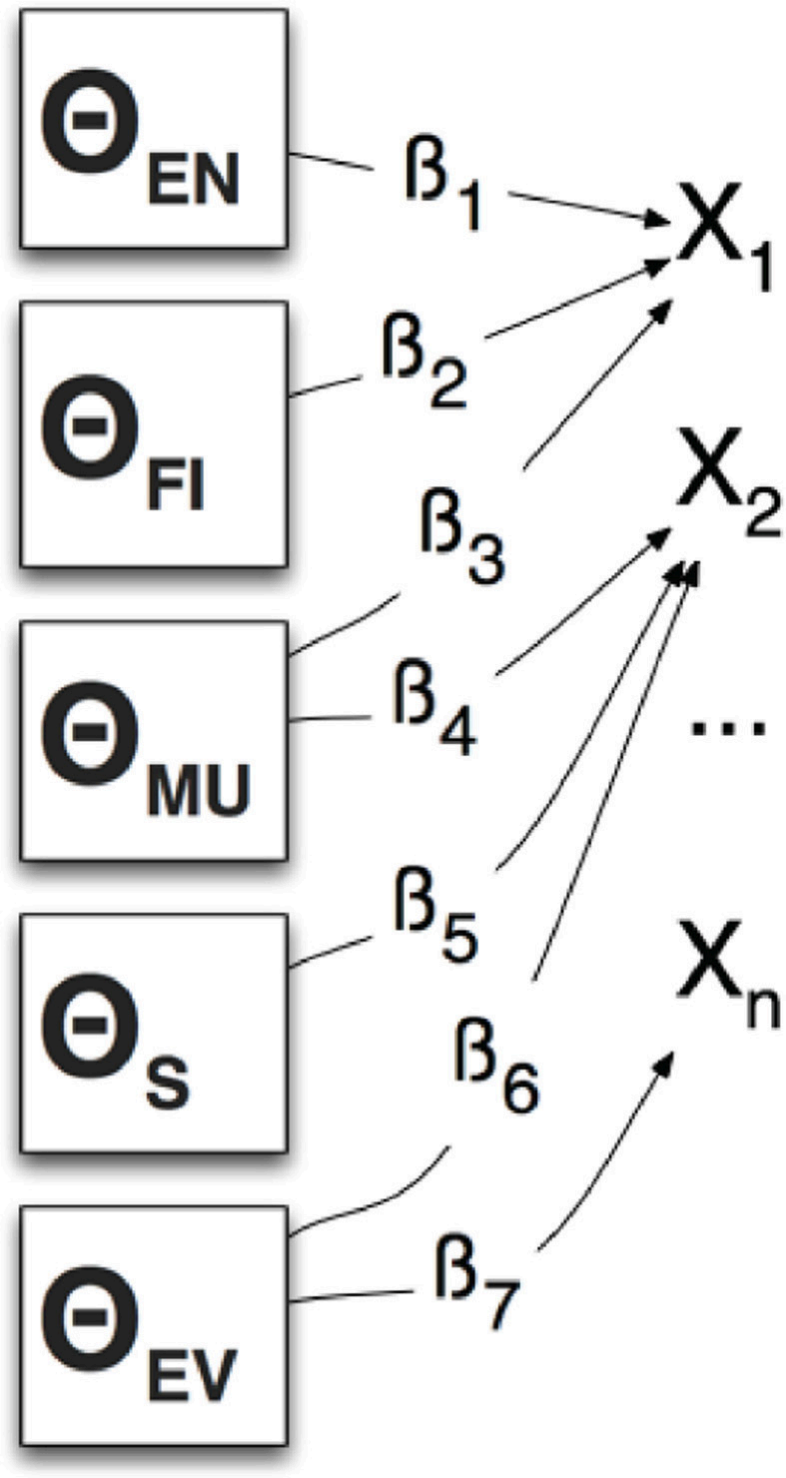
Conditional distribution of *xs* given θ.

Figure 4 presents a directed graph representation of a multivariate model with parameters that specify conditional distributions of *x*_*j*_ (an instance of a selected CPS dialog response) given θ. The β parameters can represent the “nature and strengths of the relationship” between an *x*_*j*_ and the associated latent variable θ. In this way we can express the relationship between latent variables in our model and the dialog selection evidence using conditional probability tables (CPT) (Mislevy et al., 2014).

##### 2.4.1.7. Conditional probability tables

In our Bayesian example, the *p*(*H*|*E*), or likelihood, was a function of the composition of the bins. In our application of Bayes' rule to the game prediction we will use a conditional probability table for our likelihood term instead. An example of a CPT is shown in Table 2. This table was built to provide a modest weighting that indicates a slightly higher likelihood that users will pick responses aligned with their latent variable. Using this table we can explicitly model the type of evidence (high/medium/low performance level, designated by research tagging) which is along the row and the hypothesized performance level of the latent variable (low/medium/high) along the column. Said another way, this table illustrates that if a participant's latent variable is low (row 1) then there is a slightly higher likelihood (.4) that they will select a low tagged response option instead of a medium/med or high level (.3). In practice, there could be a unique CPT created for each item/conversation prompt instance. These unique CPTs might be derived empirically through statistical analysis or could be built using expert judgement. This would allow researchers to fine tune the likelihoods based on the particular item content/difficulty.

**Table 2:** Conditional probability table (CPT).

**θ ∣ *X*_*i*_**	**Low**	**Med**	**High**
θ_*low*_	0.4	0.3	0.3
θ_*med*_	0.3	0.4	0.3
θ_*high*_	0.3	0.3	0.4

##### 2.4.1.8. Evidence tracing

In our Bayesian widget selection example, we presented two possible hypotheses: either the widget came from bin 1 or 2. For the CPS game, we are presented with a response that indicates sub-skill (*ss*_*i*_) evidence at a particular performance level. As we trace a student's selections we are maintaining three possible hypotheses about the participants latent variable per each sub-skill, viz.

1. Hypothesis: $\theta_{ss_{i}}^{high}$, Given the evidence to date, the player has a high level for this sub-skill
2. Hypothesis: $\theta_{ss_{i}}^{med}$, Given the evidence to date, the player has a medium level for this sub-skill
3. Hypothesis: $\theta_{ss_{i}}^{low}$, Given the evidence to date, the player has a low level for this sub-skill

**Figure 5:**
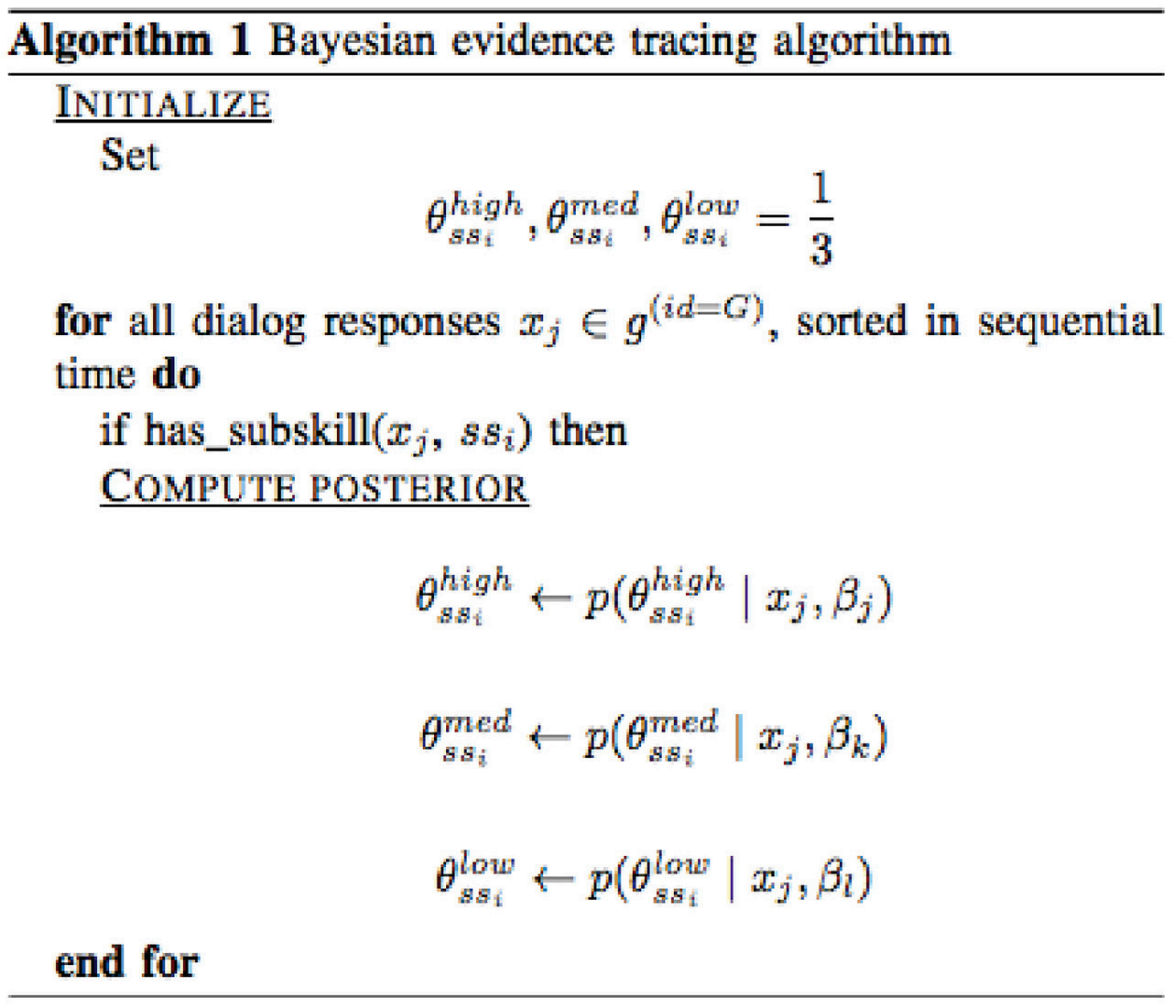
Bayesian evidence tracing algorithm.

For each game (G=game_id) then, our algorithm for computing probabilities for the performance levels of a particular sub-skill *ss*_*i*_ is presented in Figure 5.

In the initialize step, we set the prior for all hypotheses about a student's sub-skill level at $\frac{1}{3}$, since we have no other evidence. For each dialog response, if it was tagged for the sub-skill then we will recompute the posterior for each hypothesis by incorporating the new evidence. The β value used for the likelihood will be based on a CPT lookup that considers which table is being used for which dialog/response pairing and also what level the skill was tagged with. In our initial application we used the same CPT for all evidence (Table 2) but in our future work we intend to work with the dialog content authors to fine tune the application of CPTs based on a more refined judgement of distributions. We demonstrate the results of our tracing in section 3.1.

### 2.5. Study execution

We recruited a total of 159 middle school children to play the game. The game was accompanied with a research survey containing personality and background questions. The survey data included age, gender, grades, technology use, and personality facets. This study was reviewed and approved by an independent IRB, the Western Institutional Review Board and carried out in accordance with the Helsinki Declaration. The following steps were approved by the IRB above and were carried out as follows. We recognize 159 samples is rather small and could lead to sample size variation. A second study run is planned with 500 participants and our intention is to perform the same analysis on that data to see how well results concur.

#### 2.5.1. Method of subject identification and recruitment

Prospective subjects were recruited using paper and online advertisements for the study. The recruitment materials encouraged interested parties to visit a secure, publically accessible website with basic information about the study, www.stemstudies.com. Upon arriving at the website, the prospective subjects had the option to complete the consent process and play the game, and then complete the surveys. The game began by asking the visitor for their age to ensure they are eligible to participate.

#### 2.5.2. Process of consent

This study only collected personally identifiable information as required for fulfillment of the informed consent obligations for the study. This included a username for the child, an email address for the parent, and a name for the parent. Before starting the game, the child created a username, agreed to the informed consent, and provided their parent's email address. After pressing the submit button, the parents were sent an e-mail with background information on the study and a link to the informed consent workflow. Once on the website, the parent will need to provide an email address, first name, last name, and consent to the informed consent document. The informed consent and related materials about the website were available on every page's footer. The parent received a follow-up e-mail 24 h later informing them that they consented to their child's use of the website. This helped ensure that the parent actually consented to the child's use of the site and allows the parent to revoke consent.

On average, the participants spent around 30 min playing the game. We are currently performing a second run of the study that recruits 500 participants using Amazon Mechanical Turk. In that run we are also including a few more instruments in addition to the game play:

- A pre-survey and post-survey (demographics, background questions).
- A collaborative problem solving questionnaire.
- A situational judgment task assessment involving workplace behaviors relating to collaboration and problem-solving.
- A HEXACO personality assessment. HEXACO is a six-factor structure of personality-descriptive adjectives (Ashton et al., 2004).

### 2.6. Postgame analysis

In the postgame analysis, we extracted the raw conversation flow logs from the game and transformed the data to align with the skill/level tagging data provided by the ACT holistic framework researchers. We then used these data to address the following research question:

“Given the raw data of selected dialog responses across various games played by the students, can we meaningfully group patterns of selections into clusters that may represent different levels of CPS skill evidence?”

Mislevy et al. (2014) demonstrated how traditional assessment approaches relate to emerging techniques for synthesizing the evidence we outlined in our research question. In particular they demonstrate how the models/methods of psychometrics can be leveraged in game-based assessments to collect evidence about aspects of a game player's activities and capabilities.

“Exploratory data analysis (particularly visualization and hypothesis generation tools) and educational data mining techniques (including recent methods such as unsupervised neural network modeling and …cluster analysis, latent class analysis, and multidimensional scaling) can identify associations among observable features of performance that suggest new student-model variables …Educational data mining is the process of extracting patterns from large data sets to provide insights into instructional practices and student learning. It can often be employed for exactly the tasks of evidence identification: feature extraction based on patterns in data …

Bauckhage and colleagues also discussed the challenges stemming from a similar research question with respect to clustering game behavior data (Bauckhage et al., 2015).

“the proliferation of behavioral data poses the problem of how to derive insights therefrom. Behavioral data sets can be large, time-dependent and high-dimensional. Clustering offers a way to explore such data and to discover patterns that can reduce the overall complexity of the data. Clustering and other techniques for player profiling and play style analysis have, therefore, become popular in the nascent field of game analytics. However, the proper use of clustering techniques requires expertise and an understanding of games is essential to evaluate results”

Based on this and other related research (Kerr et al., 2011; Orkin and Roy, 2011; Smith, 2011; Canossa, 2013; Li et al., 2013), it was evident that a machine learning-based, clustering methodology would be useful to explore patterns within our game dialog selection data. In particular we demonstrate an application of game-related, k-means clustering [as reported in other related research (Thurau and Bauckhage, 2010)] vs. other types reported such as Linear Discriminant Analysis (LDA) (Gow et al., 2012) or Mixture Model clustering (Teófilo and Reis, 2013).

#### 2.6.1. Extract

The log data file that is extracted from the game is outlined in Table 1. As we can see, the log collects the presentation of a dialog tree prompt to the user in a game as row type “P.” The prompt presented is recorded in the column “prompt_id.” Row type “R” records the response selected by the user in the game for the prompt row immediately preceding it in the log. This raw game log file contained the game session log for several game instances.

#### 2.6.2. Transform

As we mentioned in our Bayesian workflow, our next step was to flatten this representation so that the prompt and the response rows were combined into a single record. Additionally, we also filtered out data rows that were known to be game developer “user_ids” so that we were only looking at data from actual subjects. There were also prompt rows followed by some in game action. So instead of a response to that prompt, the user had done something that subsequently caused another prompt to appear. Since there was no response to that initial prompt, it, along with the following action, were also filtered out. The N count for this analysis was 159 unique games.

#### 2.6.3. k-means methodology

The methodology we followed involved these steps:

- Extract raw conversation flow game log from a set of played games
- Transform the conversation flow into a flattened file that combines prompts and responses, and filter out any potential developer gameplay data
- Encode each game as a single row in a 1-Hot encoding of selected dialog responses
- Translate the 1-Hot encoding into 5 datasets corresponding to evidence acquired on all 5 CPS domains
- Perform basic scoring of each game on the 5 CPS domains
- Perform k-means clustering (Steinhaus, 1956) of game domain scores
- Present summary and results of clustering.

#### 2.6.4. Encode/translate

Taking the flattened prompt/response data we encoded each game as a single row in a 159x286 matrix. The number of rows is the *N* count and the number of columns are the 3 identifiers (session, user, game) plus the 283 potential, selectable dialog responses (*D* = 283). We encoded a “1” if the user selected the identified response at any time during the game. It should be noted that several of the dialog sub-trees can allow a user to loop back through the tree within a single game. If the user selected a particular response more than once in a game we still recorded the selection with a single “1.” Otherwise, if the user never selected a particular response during the game the encoding for that column was “0.” Each of the unique dialog prompt/response combinations were coded based on the 5 domains as defined in the CPS game data section.

Given this mapping, we were able to create 5 domain evidence matrix variations on the 1-Hot matrix where we substituted the 1,0 with a value of 0,1,2,3 corresponding to the evidence values (no/low/med/high evidence). See Figure 2.

#### 2.6.5. Score

Given the 5 domain evidence matrices (as a variation from the 1-Hot encoding) we could then score a game on each of the 5 domains by a simple summing of evidence across each response feature.

$$score^{FI}=\sum_{d=1}^{D}x_{d}^{FI}$$

$$score^{MU}=\sum_{d=1}^{D}x_{d}^{MU}$$

$$score^{EN}=\sum_{d=1}^{D}x_{d}^{EN}$$

$$score^{EV}=\sum_{d=1}^{D}x_{d}^{EV}$$

$$score^{S}=\sum_{d=1}^{D}x_{d}^{S}$$

We then reformed the scores into a domain score matrix 159x8 where the rows = N and the columns were the 3 identifiers (session, user, game) plus the 5 summed evidence score for each domain as show in Table 3.

**Table 3:** Scores matrix.

**Session_id**	**User_id**	**Game_id**	**FI_Score**	**MU_Score**	**EN_Score**	**EV_Score**	**S_Score**
46	33	211	47	10	26	3	7
57	38	310	39	21	31	4	9
…							

#### 2.6.6. Cluster

Using this derived score matrix we then performed an unsupervised learning k-means clustering of the data using the Graphlab-Create library^2^. We selected the *K*-value based on the following heuristic: $K=\sqrt{N/2.0}=8$ clusters

#### 2.6.7. K exploration

Starting with *K* = 8 based on the heuristic value, we continued to evaluate additional potential *K*-value assignments. The k-means implementation of Graphlab-Create uses the k-means++ algorithm for initial choice of cluster centers. This results in some randomization and variance of cluster assignment with each building of the model. As we visualized the data points with the assignment of the *K* = 8 clusters we noticed similar patterns between several of the clusters. In particular, there appeared to be overlap between 4 sets of 2. This indicated that a 4 cluster assignment may be more appropriate.

We decided to build the model numerous times with a *K*-value of 8 and compare cluster assignments between these model building runs. We saw that row assignment from the initial cluster assignment didn't always result in classification to the same cluster as on a subsequent build of the model. Sorting the data on the first model build and looking at the cluster classification across the next two builds of the model, we saw some of the same assignments. We subsequently chose *K* = 6 and performed the same multiple run build of the model. Drift was somewhat less, but not significantly so. Setting *K* = 4 and building the model several times showed much less variance in cluster assignment. There was still some drift, but it was significantly less than what we saw with a *K* = 8 and in general cluster assignments persisted across multiple builds of the model even with randomly chosen initial centers.

#### 2.6.8. K-NN query by game id

In addition to the k-means model, we also built a K-Nearest Neighbor (K-NN) model (Arya et al., 1998) using Graphlab-Create which allows us to go back and query the data for games that were similar to a selected game id using a cosine similarity distance metric.

#### 2.6.9. Mixture model methodology

There are drawbacks to using the k-means clustering algorithm:

- assumes a specific shape of cluster distributions (spherically symmetric)
- only provides hard assignments to one of the possible clusters.

k-means can be understood as a specific instance of a more generic approach to clustering that is defined by analyzing a mixture of distributions that can be computed using an Expectation Maximization (EM) algorithm (McLachlan and Basford, 1988). Following the same methodology we outlined above to derive our data frame of CPS dialog scores, we re-ran clustering using a mixture of Gaussians approach. This allows us to:

- learn the means and co-variances of each Gaussian distribution (asymmetric, elliptical cluster shapes)
- compute soft assignments to clusters using a Bayesian calculation.

In particular, the EM algorithm works by iteratively running an E-step and M-step where:

1. E-step: estimates cluster responsibilities given current parameter estimates
  $${\hat{r}}_{ik}=\frac{{\hat{\pi }}_{k}N\left(x_{i}|{\hat{\mu }}_{k}{\hat{\sum }}_{k}\right)}{\sum_{j=1}^{K}\hat{\pi }N\left(x_{i}|{\hat{\mu }}_{j},{\hat{\sum }}_{j}\right)}$$
2. M-step: maximizes likelihood over parameters given current responsibilities
  $${\hat{\pi }}_{k},{\hat{\mu }}_{k},{\hat{\sum }}_{k}|\{{\hat{r}}_{ik},x_{i}\}$$

From a Bayesian perspective, the ${\hat{r}}_{ik}$ probability represents the responsibility that cluster *k* claims for observation *i* expressed as a posterior distribution. This is computed based on ${\hat{\pi }}_{k}$, the prior probability of cluster *k*, and the likelihood that observation *i* (based on a Gaussian distribution) would be assigned to cluster *k* given the mean and covariance of the distribution: $N\left(x_{i}|{\hat{\mu }}_{k},{\hat{\sum }}_{k}\right)$ divided by the normalizing constant which considers the probability over all possible clusters $\sum_{j=1}^{K}\hat{\pi }N\left(x_{i}|\hat{\mu_{j}},{\hat{\sum }}_{j}\right)$.

We implemented the code for both the E-step and M-step in Python and ran the implementation over 120 iterations using the MU, FI and EN scores. The S and EV domains were excluded based on their low information content. We also implemented a matplotlib function to plot the computed responsibilities after a specified number of iterations in order to show how the clustering evolved over time. We present those plots in the clustering results section.

## 3. Results

In the results section, we present visualizations of real-time Bayesian evidence tracing based on a participant's continuous log evidence. We also present the results from our clustering data along with views of cluster data indicators and distributions.

### 3.1. Bayesian evidence tracing results

**Figure 6:**
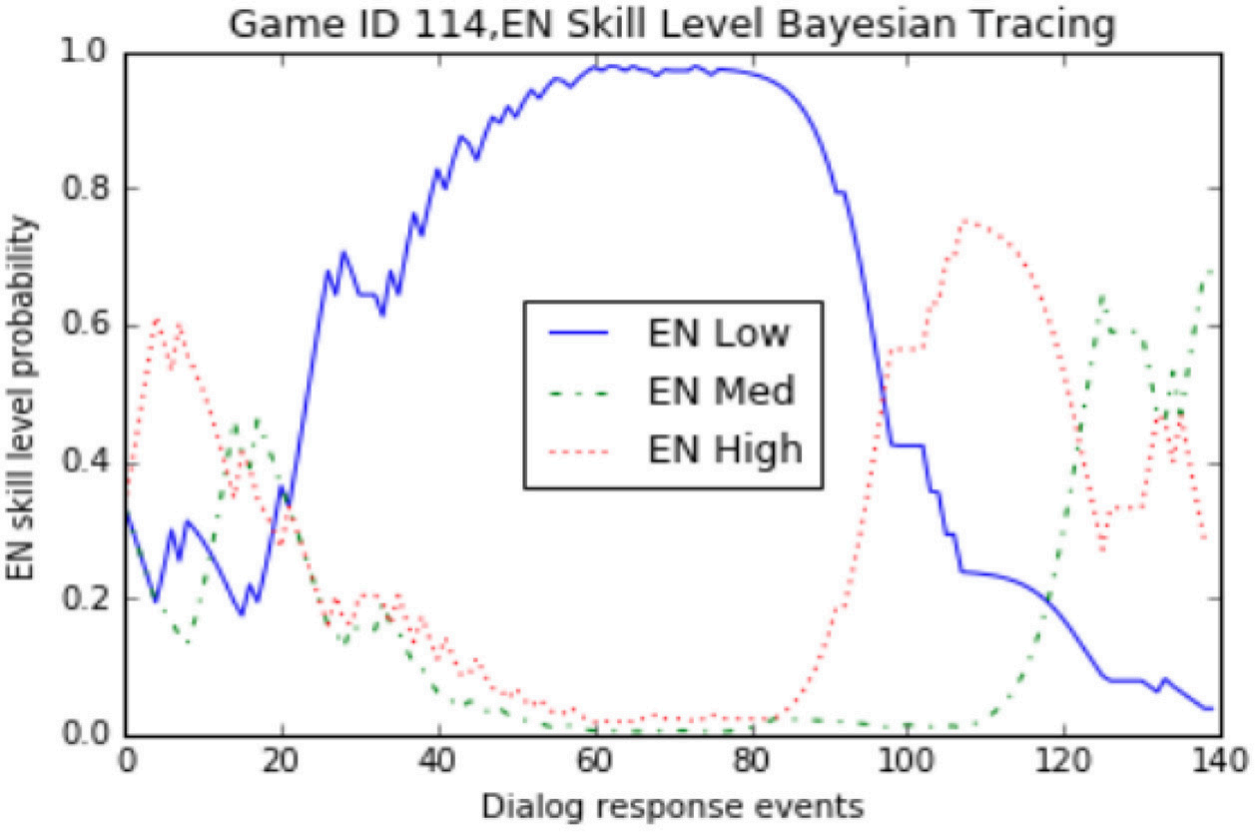
Engagement (EN) sub-skill level probability over time for a single game.

**Figure 7:**
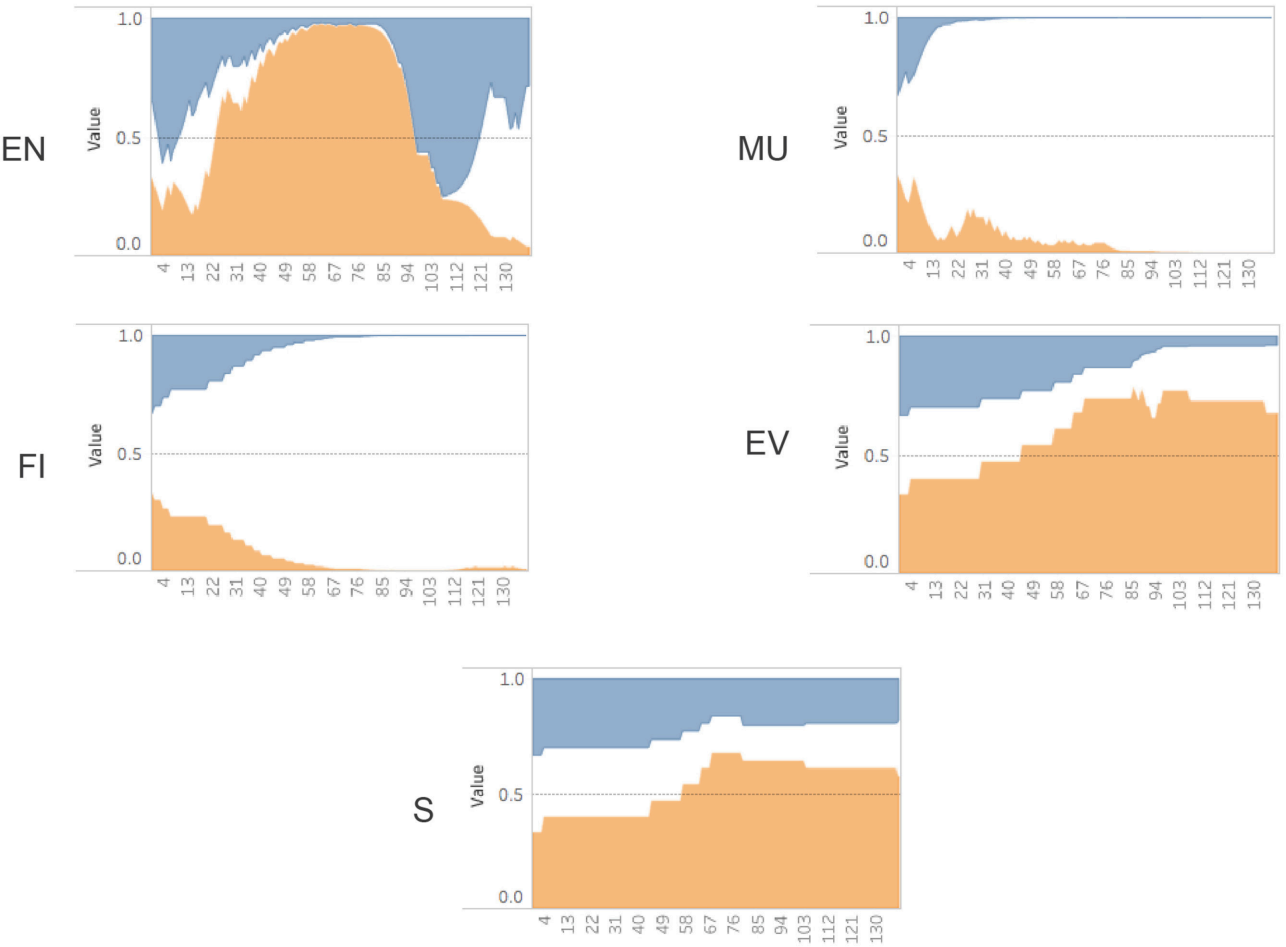
Probability (y-axis) over time (x-axis) for a single game (game id = 114) (Blue = High, White = Med, Orange = Low). Engagement (EN), Monitor Understanding (MU), Feature Identification (FI), Evaluation (EV), Strategy (S).

Our implementation of the Bayesian algorithm described in Figure 5 was done in Python using a Jupyter notebook^3^ web application. We also used the SFrame API from Graphlab-Create to manipulate the game log data^4^. In order to visualize the sub-skill probabilities over time we initially used matplotlib^5^. An example of the plot for a sample game_id = 114 can be seen in Figure 6. This graph shows the increases and decreases of the probability estimates for a participant's EN sub-skill over time. There are three lines because we are tracking each level (high/medium/low) as a separate, but linked variable. All three variables begin using a prior set at .333 and then diverge as the evidence is traced using Bayesian analysis. Additionally we used Tableau^6^ to render similar views as can be seen in Figure 7. This view allows an analyst to see the predictions of performance levels for each skill, over time, for a single game. The blue area represents a high level, the white area is medium level, and the orange area is the probability of a low level. This view uses an area of fill representation.

Looking at the evidence collected for the single game_id = 114 Figure 7, we can see the sub-skills for monitoring understanding (MU) and feature identification (FI) quickly settled on a “medium” level assessment during the first third of the total dialog response interactions. In contrast, the strategy (S) and evaluate (EV) sub-skills settled on a “low” level assessment over the final two thirds of the interactions. The engagement (EN) scores showed fairly dramatic swings between all three performance levels over time, ultimately finishing with a “medium” level assessment. If we were restricted to only looking at the final probabilities (posterior values), we wouldn't have been able to notice these real-time patterns in gameplay. Since the Bayesian Evidence Tracing algorithm is an “anytime algorithm,” we are able to directly interrogate this model at any point to determine the current estimate of a user's sub-skill probability.

### 3.2. Clustering results

As we described in our methods section, we implemented two clustering approaches, a hard clustering assignment with k-means and a soft clustering assignment using a Gaussian mixture model approach. Additionally, we implemented a K-nearest neighbor (K-NN) mechanism to lookup related games based on the clustering data. The purpose of applying these classification approaches is to look for naturally occuring groups and to determine emergent patterns of game plays or skills.

#### 3.2.1. k-means/K-NN results

**Figure 8:**
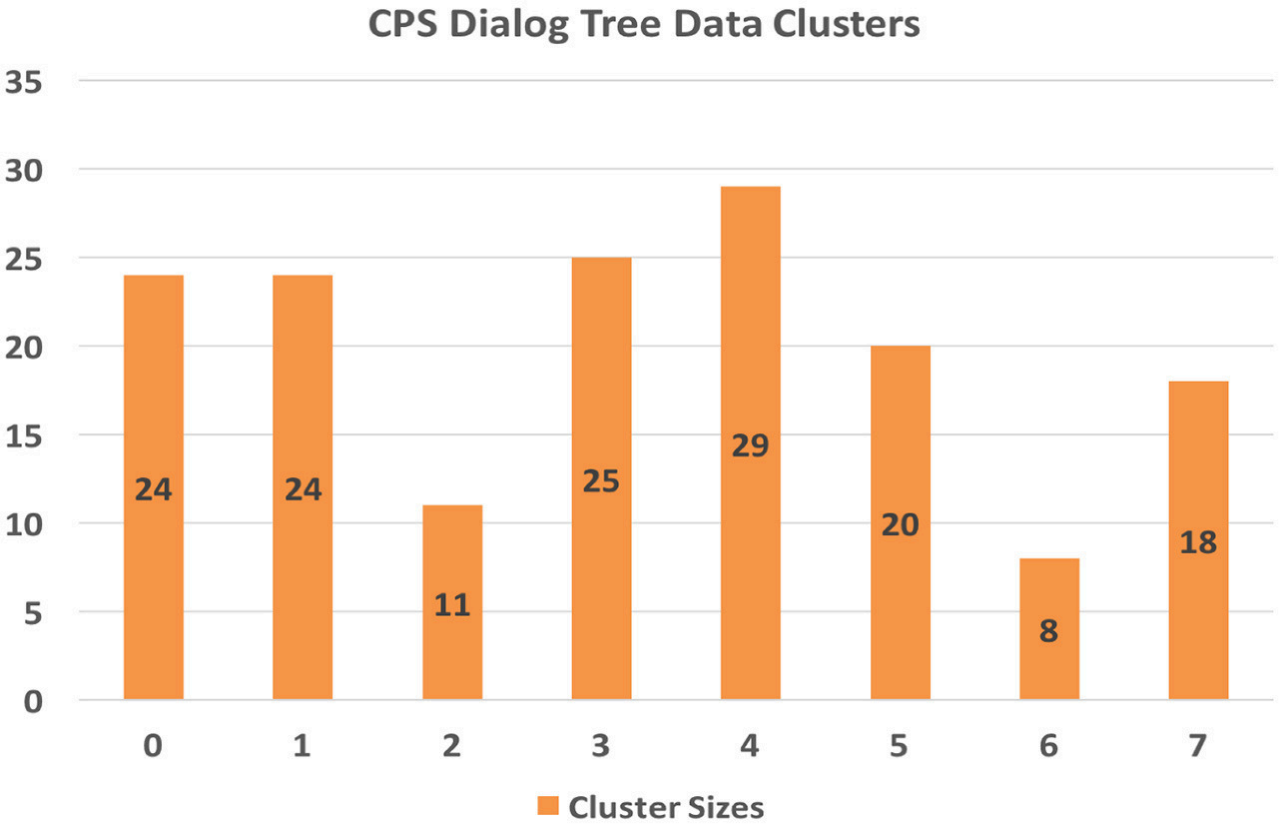
CPS data cluster counts.

The clustering model using the k-means approach yielded the game counts per cluster as shown in Figure 8.

#### 3.2.2. Cluster characteristics

Now that we have created a clustering model of the game evidence scores, we can inspect the model to see what each cluster might represent about the player/game play evidence of CPS. To that end, we can look at the mean score for each of the 5 domain areas for the members of each cluster. The score scales of the 5 domains scores vary considerably, viz. the “EV” and “S” mean scores are much smaller.

For visualization purposes, we normalized the mean scores as follows:

$$x_{new}=\frac{x-x_{min}}{x_{max}-x_{min}}$$

**Figure 9:**
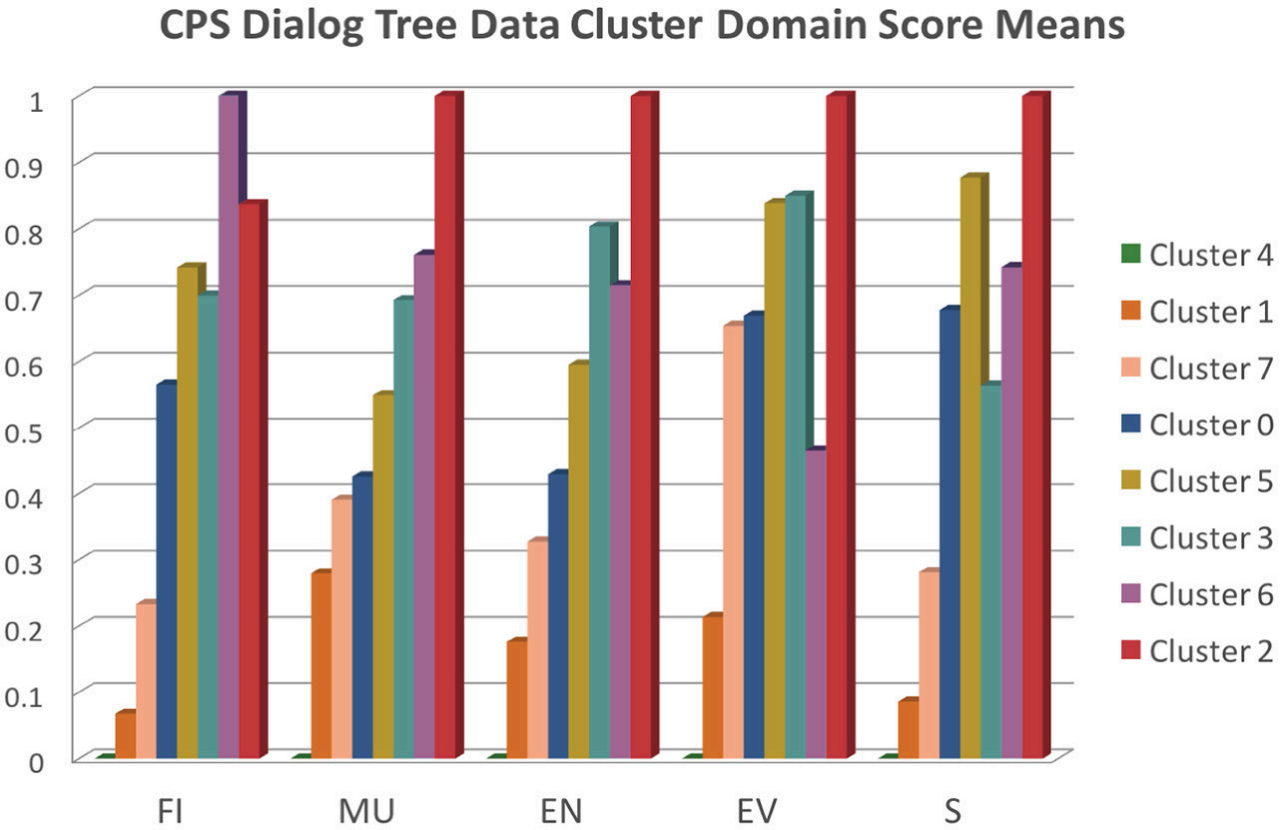
CPS data cluster domain score means.

In Figure 9 we present a graph of the normalized mean scores for each domain across all 8 clusters. We roughly sorted the clusters from left to right within each sub-skill column according to relatively increasing score means. For reference, the raw mean score for each of the sub-skills are: FI = 28, MU = 17, EN = 31, EV = 3, S = 2, and the raw standard deviation for each is: FI = 18.49129825, MU = 10.52478206, EN = 21.48635757, EV = 2.128990584, S = 3.603795468.

Cluster 2 (*N* = 11) represents the games that exhibit the highest CPS scores across nearly all domains (except for FI), whereas cluster 4 (*N* = 29) represents the games that exhibit the lowest CPS scores. Given that we didn't filter out incomplete games, i.e., games where subjects did not make it all the way through the final challenge, it is likely that cluster 4 represents many of these incomplete games. Cluster 6 (*N* = 8) game plays excelled at FI and presented very good scores across the board as well. Cluster 3 (*N* = 25) games provided a balanced set of very good scores, especially in EN and EV. Cluster 5 (*N* = 20) game plays excelled at EV and S. Cluster 1 game plays (*N* = 24) provided fairly weak evidence of CPS skills overall, whereas clusters 7 (*N* = 18) and 0 (*N* = 24) presented low to average scores.

**Figure 10:**
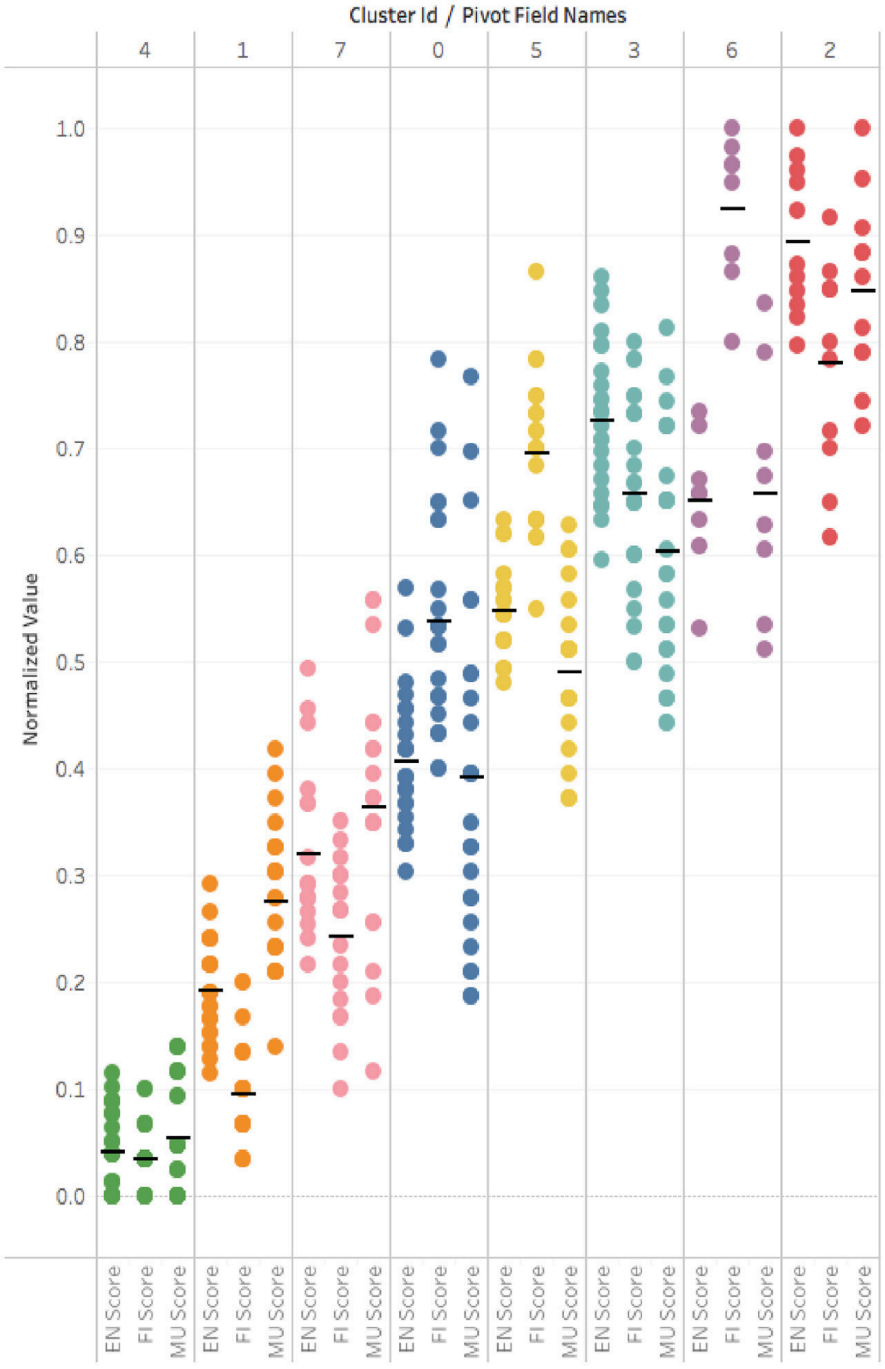
Tableau visualization of scores by cluster. Each color/column is a cluster. A dot represents a game score in CPS sub-skill {EN,FI,MU}. A black line shows a cluster mean score.

We also loaded the data into a Tableau workbook^7^ to analyze the cluster characteristics using various worksheets. In that analysis, we saw a vertical distribution of normalized scores grouped by score feature (EN, FI, MU, S, EV) for each of the 8 clusters that showed that while EN, FI, and MU features appeared to have fairly tightly grouped cluster values the features values from S, EV appeared to be much more diffuse within a cluster. As EN, FI, and MU are the important feature drivers of the cluster characteristics we looked at a similar view. That allowed us to examine the cluster distributions across a range of score groupings over EN, FI and MU. In Figure 10 we re-arrange the data to illustrate the vertical cluster scores (the black line indicates the mean) with each column as a cluster.

#### 3.2.3. K-NN query by game id

In addition to the k-means model, we also built a K-Nearest Neighbor (K-NN) model (Arya et al., 1998) using Graphlab-Create, which allows us to go back and query the data for games that were similar to the source game using a cosine similarity distance metric.

#### 3.2.4. Mixture model results

**Figure 11:**
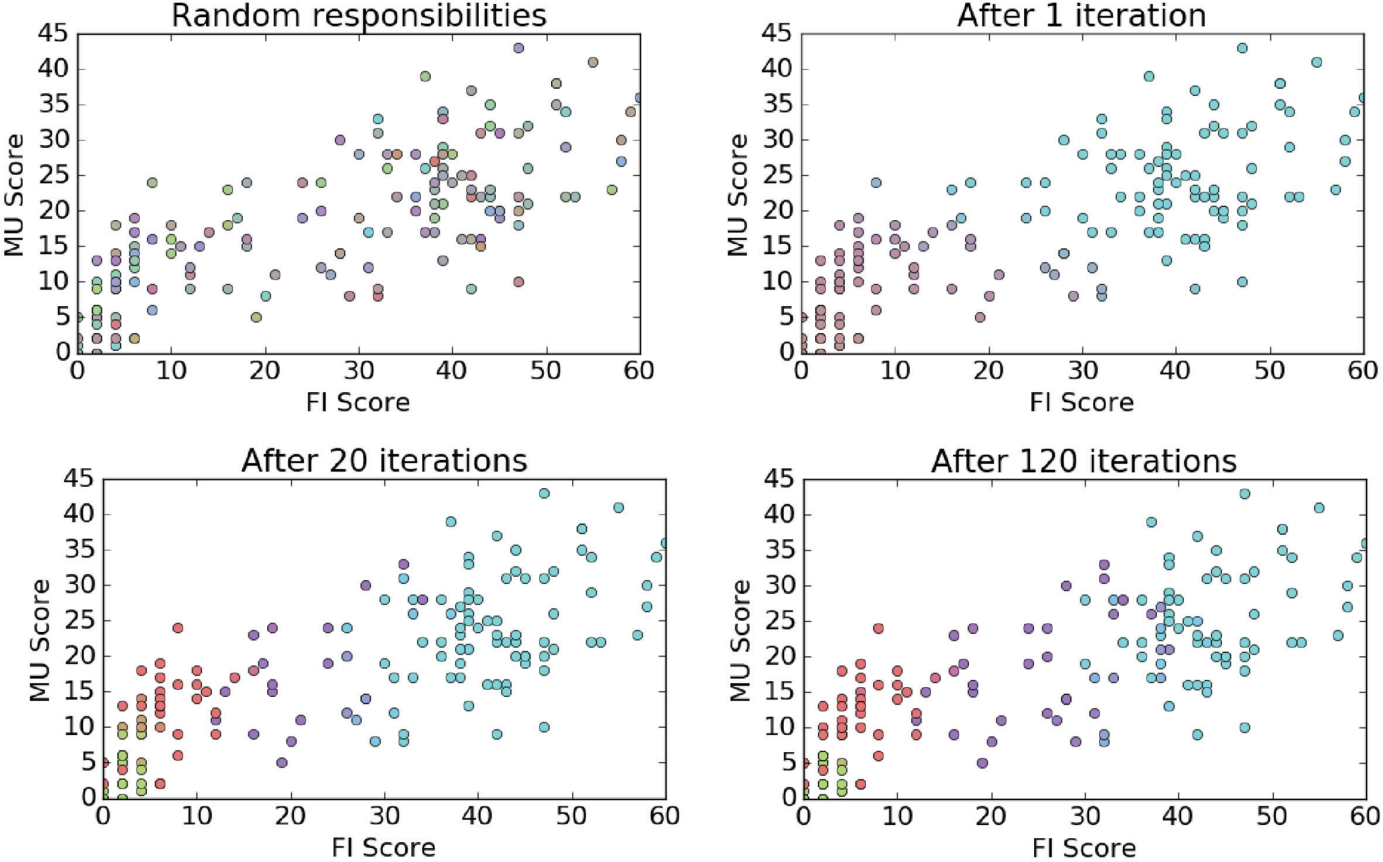
EM clustering visualization.

In Figure 11 we represent how our application of an EM algorithm learned the dialog score cluster responsibilities over a series of iterations. For 2-D visualization purposes we just show the MU/FI features. The color of each dot represents a blending of cluster probabilities.

**Figure 12:**
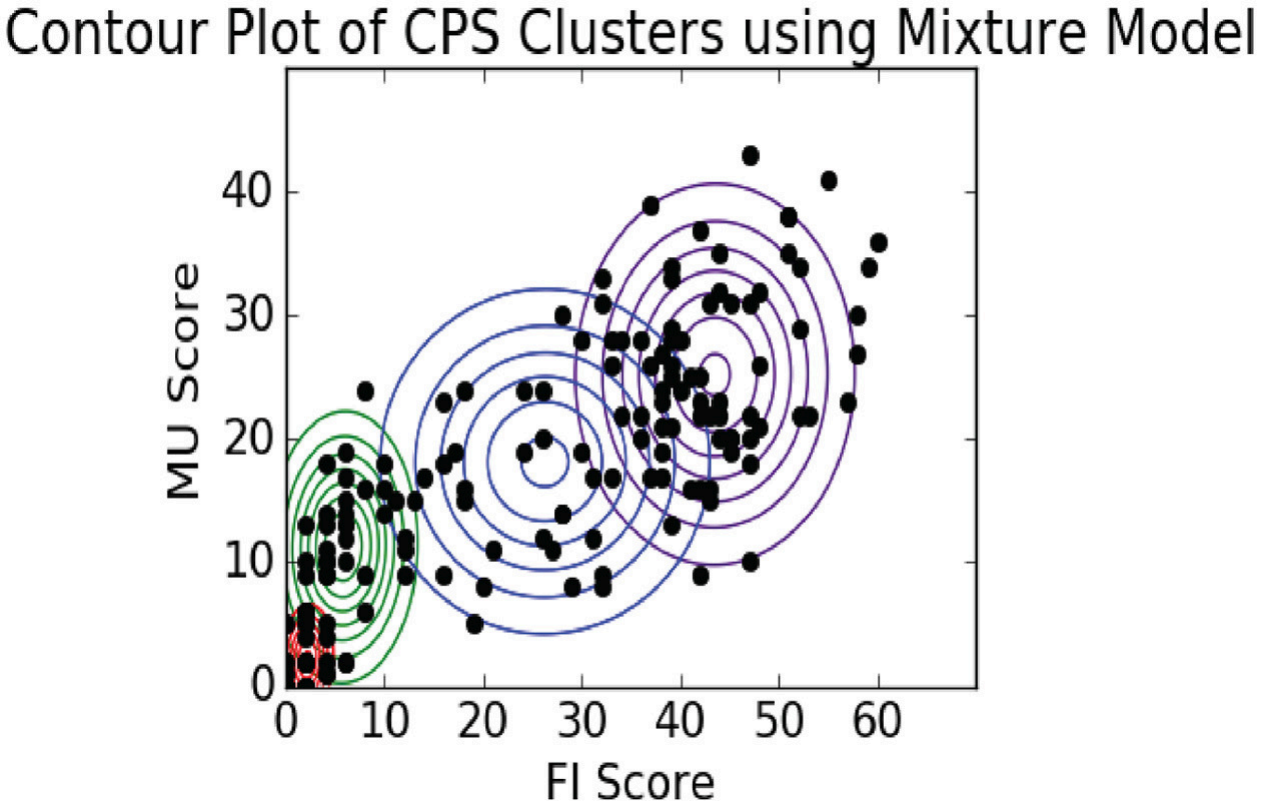
EM contour plot.

As we can see the Mixture Model approach updates the cluster distribution shapes over each iteration, effectively learning the mean and covariance of each distribution. In Figure 12 we plot the final shape of the cluster distributions (*k* = 4), again limiting this to just the MU and FI score dimensions. As we can see, this method of clustering allowed the model to learn asymmetric elliptical cluster shapes and also provided us with probabilistic assignments of each observation to any of the clusters. Thus, we are able to represent more robust cluster characterizations beyond a simple in/out hard assignment.

Our interpretation of these data is that the observations in the upper right cluster represent players that were exhaustively exploring the dialog trees which resulted in maximizing their dialog scores. The next cluster to the left represents players who were focused on getting just the data they needed in their collaboration to complete the challenges. The two far left, bottom clusters represent players that were not engaged and probably didn't play through to the final challenge.

## 4. Discussion

In this paper we have demonstrated the application of computational psychometrics to gathering insights into a participant's CPS sub-skills using evidence gathered from an online simulation/game. We showed how we can take the granular evidence gathered from the conversation flow and simulation/game activity data and map that onto our performance level estimates of latent variables, such as CPS skills. These higher level constructs are driven by CPS subject matter expert tagging and tunable conditional probability tables. This methodology creates a model that can be inspected at any time during the game to provide a probability-based estimate of participant ability. As we move forward with this work we can use this model to start to build more sophisticated simulation/game interactions that could change adaptively, based on our real-time estimate of ability. For example, if we see participants are showing evidence of low feature identification we can add cues/tips to help them in this facet of interaction.

While the real-time Bayesian evidence tracing has proven useful in generating actionable insights for an individual participant during a game, our clustering work reported here has addressed our need to also compare across games. Our application of k-means gave us the ability to quickly characterize all games in the study and to group similar gameplays with each other, thus yielding different game profiles. Using K-NN we are able to treat these clusters as queryable sets that allow us to find participants that had similar evidence patterns of CPS sub-skills. In applying our Gaussian mixture model we were able to generate a more flexible cluster characterization of each game that can allow for partial cluster membership in more than 1 game profile.

We are working on the next iteration of our Circuit Runner game using the methods and results we have reported here. In our future work we are considering the integration of Bayesian evidence tracing with an application of adaptive conversation flows. We are also incorporating new instruments that will provide more demographics/data on the participants, such as a HEXACO assessment of personality and the results of a CPS questionnaire. We are also considering human-human CPS interaction scenarios that could feature scripted or open-ended conversations.

## Author contributions

All authors listed have made a substantial, direct and intellectual contribution to the work, and approved it for publication.

### Conflict of interest statement

The authors declare that the research was conducted in the absence of any commercial or financial relationships that could be construed as a potential conflict of interest.

## Footnotes

^1^https://cpsgame.stemstudies.com

^2^https://turi.com/products/create/

^3^http://jupyter.org

^4^https://turi.com/products/create/

^5^http://matplotlib.org

^6^http://www.tableau.com

^7^http://www.tableau.com

## References

[B1] Arya S. Mount D. M. Netanyahu N. S. Silverman R. Wu A. Y. (1998). An optimal algorithm for approximate nearest neighbor searching fixed dimensions. JACM 45, 891–923. 10.1145/293347.293348

[B2] Ashton M. C. Lee K. Perugini M. Szarota P. De Vries R. E. Di Blas L.. (2004). A six-factor structure of personality-descriptive adjectives: solutions from psycholexical studies in seven languages. J. Personal. Soc. Psychol. 86:356. 10.1037/0022-3514.86.2.356 14769090

[B3] Bauckhage C. Drachen A. Sifa R. (2015). Clustering game behavior data. IEEE Trans. Comput. Intel. AI Games 7, 266–278. 10.1109/TCIAIG.2014.2376982

[B4] Bayes M. Price M. (1763). An essay towards solving a problem in the doctrine of chances. by the late rev. mr. bayes, frs communicated by mr. price, in a letter to john canton, amfrs. Philos. Trans. 53, 370–418. 10.1098/rstl.1763.0053

[B5] Bazaldua D. Khan S. von Davier A. A. Hao J. Liu L. Wang Z. (2015). On convergence of cognitive and non-cognitive behavior in collaborative activity, in Proceedings of the 8th International Conference on Educational Data Mining (Madrid), 496–499.

[B6] Bergner Y. Andrews J. J. Zhu M. Kitchen C. (2015). Agent-based modeling of collaborative problem solving, in Paper presented at the 10th Annual INGRoup Conference (Pittsburgh, PA).

[B7] Camara W. O'Connor R. Mattern K. Hanson M. A. (2015). Beyond Academics: A Holistic Framework for Enhancing Education and Workplace Success. Act research report series. ACT Inc.

[B8] Canossa A. (2013). Meaning in gameplay: filtering variables, defining metrics, extracting features and creating models for gameplay analysis, in Game Analytics, eds Seif El-Nasr M. Drachen A. Canossa A. (London: Springer), 255–283.

[B9] Corbett A. T. Anderson J. R. (1994). Knowledge tracing: modeling the acquisition of procedural knowledge. User Model. User Adap. Inter. 4, 253–278. 10.1007/BF01099821

[B10] Davey T. Ferrara S. Shavelson R. Holland P. Webb N. Wise L. (2015). Psychometric Considerations for the Next Generation of Performance Assessment. Washington, DC: Center for K-12 Assessment & Performance Management, Educational Testing Service.

[B11] Desmarais M. C. Baker R. S. (2012). A review of recent advances in learner and skill modeling in intelligent learning environments. User Model. User Adap. Inter. 22, 9–38. 10.1007/s11257-011-9106-8

[B12] Gow J. Baumgarten R. Cairns P. Colton S. Miller P. (2012). Unsupervised modeling of player style with lda. IEEE Trans. Comput. Intel. AI Games 4, 152–166. 10.1109/TCIAIG.2012.2213600

[B13] Griffin P. McGaw B. Care E. (2012). Assessment and Teaching of 21st Century Skills. Dordrecht: Springer.

[B14] Hao J. Liu L. von Davier A. A. Kyllonen P. Kitchen C. (2016). Collaborative problem solving skills versus collaboration outcomes: findings from statistical analysis and data mining, in Proceedings of the 9th International Conference on Educational Data Mining (Raleigh, NC).

[B15] He Q. von Davier M. Greiff S. Steinhauer E. W. Borysewicz P. B. (2017). Collaborative problem solving measures in the programme for international student assessment (pisa), in Innovative Assessment of Collaboration, eds von Davier A. A. Zhu M. Kyllonen P. C. (Cham: Springer), 95–111.

[B16] Kerr D. (2015). Using data mining results to improve educational video game design. J. Educ. Data Mining 7, 1–17. Available online at: http://jedm.educationaldatamining.org/index.php/JEDM/article/view/JEDM048

[B17] Kerr D. Chung G. K. (2012). Identifying key features of student performance in educational video games and simulations through cluster analysis. J. Educ. Data Mining 4, 144–182. Available online at: http://jedm.educationaldatamining.org/index.php/JEDM/article/view/25

[B18] Kerr D. Chung G. K. Iseli M. R. (2011). The Feasibility of Using Cluster Analysis to Examine Log Data from Educational Video games. Cresst report 790. National Center for Research on Evaluation, Standards, and Student Testing (CRESST).

[B19] Khan S. (2015). Multimodal Behavioral Analytics for Intelligent Training and Assessment Systems. The rutgers university.

[B20] Khan S. Cheng H. Kumar R. (2013). A hierarchical behavior analysis approach for automated trainee performance evaluation in training ranges, in Proceedings of the 7th International Conference on Augmented Cognition, eds Schmorrow D. D. Fidopiastis C. M. (Berlin; Heidelberg: Springer), 60–69.

[B21] LaMar M. M. (2014). Models for Understanding Student Thinking Using Data from Complex Computerized Science Tasks. PhD thesis, University of California, Berkeley.

[B22] Levy R. (2014). Dynamic Bayesian Network Modeling of Game Based Diagnostic Assessments. Cresst report 837. National Center for Research on Evaluation, Standards, and Student Testing (CRESST). 10.1080/00273171.2019.1590794 30942094

[B23] Li H. Munoz-Avila H. Ke L. Symborski C. Alonso R. (2013). Discovery of player strategies in a serious game, in Proceedings of the First AAAI Conference on Human Computation and Crowdsourcing (Palm Springs, CA).

[B24] McLachlan G. J. Basford K. E. (1988). Mixture Models. Inference and Applications to Clustering. New York: Dekker: Statistics: Textbooks and Monographs.

[B25] Mislevy R. J. Oranje A. Bauer M. I. von Davier A. A. Hao J. Corrigan S. (2014). Psychometric Considerations in Game-Based Assessment. GlassLab Report. GlassLab Research, Institute of Play.

[B26] Mislevy R. J. Steinberg L. S. Almond R. G. (2003). Focus article: on the structure of educational assessments. Measure. Interdiscipl. Res. Perspect. 1, 3–62. 10.1207/S15366359MEA0101_02

[B27] Mislevy R. J. Steinberg L. S. Almond R. G. Lukas J. F. (2006). Concepts, terminology, and basic models of evidence-centered design, in Automated Scoring of Complex Tasks in Computer-Based Testing, eds Williamson D. M. Mislevy R. J. Bejar I. I. (Mahwah, NJ: Routledge), 15–47.

[B28] NCES (2017). Collaborative Problem Solving: Considerations for the National Assessment of Educational Progress. NCES.

[B29] Oliveri M. E. Lawless R. Molloy H. (2017). A literature review on collaborative problem solving for college and workforce readiness. ETS Res. Rep. Ser. [Epub ahead of print]. 10.1002/ets2.12133

[B30] Orkin J. Roy D. (2011). Semi-automated dialogue act classification for situated social agents in games, in Agents for Games and Simulations II, ed Dignum F. (Berlin: Springer), 148–162.

[B31] Paulhus D. L. Vazire S. Robins R. W. Fraley R. Krueger R. F. (2007). The self-report method. Handb. Res. Methods Personal. Psychol. 1, 224–239.

[B32] PISA OECD. (2013). Results: Excellence Through Equity: Giving Every Student the Chance to Succeed. (Vol. ii). PISA, OECD.

[B33] Robins R. W. John O. P. (1997). The quest for self-insight: theory and research on accuracy and bias in self-perception. Handb. Pers. Psychol. 649–679. 10.1016/B978-012134645-4/50026-3

[B34] Romero C. González P. Ventura S. Del Jesús M. J. Herrera F. (2009). Evolutionary algorithms for subgroup discovery in e-learning: a practical application using moodle data. Exp. Syst. Appl. 36, 1632–1644. 10.1016/j.eswa.2007.11.026

[B35] Sánchez J. Olivares R. (2011). Problem solving and collaboration using mobile serious games. Comput. Educ. 57, 1943–1952. 10.1016/j.compedu.2011.04.012

[B36] Shute V. J. Hansen E. G. Almond R. G. (2008a). You can't fatten a hog by weighing it-or can you? Evaluating an assessment for learning system called aced. Int. J. Artif. Intel. Educ. 18, 289–316. Available online at: https://content.iospress.com/articles/international-journal-of-artificial-intelligence-in-education/jai18-4-02

[B37] Shute V. J. Ventura M. Bauer M. Zapata-Rivera D. (2008b). Monitoring and fostering learning through games and embedded assessments. ETS Res. Rep. Ser. 2008, i–32. 10.1002/j.2333-8504.2008.tb02155.x

[B38] Smith T. S. (2011). Unsupervised Discovery of Human Behavior and Dialogue Patterns in Data from an Online Game. PhD thesis, Massachusetts Institute of Technology.

[B39] Soller A. Stevens R. (2007). Applications of stochastic analyses for collaborative learning and cognitive assessment, in Advances in Latent Variable Mixture Models, eds Hancock G. R. Samuelsen K. M. (Charlotte, NC: IAP), 217–253.

[B40] Steiner I. (1972). Group Processes and Productivity. Cell Biology. New York, NY: Academic Press.

[B41] Steinhaus H. (1956). Sur la division des corp materiels en parties. Bull. Acad. Polon. Sci. 1:801.

[B42] Sung H.-Y. Hwang G.-J. (2013). A collaborative game-based learning approach to improving students' learning performance in science courses. Comput. Educ. 63, 43–51. 10.1016/j.compedu.2012.11.019

[B43] Teófilo L. F. Reis L. P. (2013). Identifying player\'s strategies in no limit texas hold\'em poker through the analysis of individual moves. arXiv preprint arXiv:1301.5943.

[B44] Thurau C. Bauckhage C. (2010). Analyzing the evolution of social groups in World of Warcraft®, in Computational Intelligence and Games (CIG), 2010 IEEE Symposium on (Copenhagen: IEEE), 170–177.

[B45] VanLehn K. (2008). Intelligent tutoring systems for continuous, embedded assessment, in The Future of Assessment: Shaping Teaching and Learning, ed Dwyer C. A. (New York: Routledge), 113–138. Available online at: http://www.public.asu.edu/~kvanlehn/Not%20Stringent/PDF/06Dwyer.pdf

[B46] von Davier A. A. (2017). Collaborative educational assessments [special issue]. J. Educ. Measure. 54, 1–141. 10.1111/jedm.12129

[B47] von Davier A. A. (2015). Virtual and collaborative assessments: Examples, implications, and challenges for educational measurement, in Invited Talk Presented at the Workshop on Machine Learning for Education, International Conference of Machine Learning (Lille).

[B48] von Davier A. A. Halpin P. F. (2013). Collaborative problem solving and the assessment of cognitive skills: psychometric considerations. ETS Res. Rep. Ser. 2013, i–36. 10.1002/j.2333-8504.2013.tb02348.x

[B49] von Davier A. A. van der Schaar M. Baraniuk R. (2016). Workshop on machine learning for education, in International Conference of Machine Learning (New York, NY).

[B50] von Davier A. A. Zhu M. Kyllonen P. C. (2017). Innovative Assessment of Collaboration. Cham: Springer.

[B51] Zhang M. Hao J. Li C. P. D. (2015) Classification of writing styles using keystroke logs: a hierarchical vectorization approach, in Paper Presented at International Meeting of the Psychometric Society (Beijing).

